# Artificial Intelligence Advancements in Oncology: A Review of Current Trends and Future Directions

**DOI:** 10.3390/biomedicines13040951

**Published:** 2025-04-13

**Authors:** Ellen N. Huhulea, Lillian Huang, Shirley Eng, Bushra Sumawi, Audrey Huang, Esewi Aifuwa, Rahim Hirani, Raj K. Tiwari, Mill Etienne

**Affiliations:** 1 School of Medicine, New York Medical College, Valhalla, NY 10595, USArhirani2@student.nymc.edu (R.H.);; 2 Barshop Institute, The University of Texas Health Science Center, San Antonio, TX 78229, USA; 3 Graduate School of Biomedical Sciences, New York Medical College, Valhalla, NY 10595, USA; 4 Department of Neurology, New York Medical College, Valhalla, NY 10595, USA

**Keywords:** cancer, oncology, artificial intelligence, machine learning, deep learning, nanomedicine, social determinants of health

## Abstract

Cancer remains one of the leading causes of mortality worldwide, driving the need for innovative approaches in research and treatment. Artificial intelligence (AI) has emerged as a powerful tool in oncology, with the potential to revolutionize cancer diagnosis, treatment, and management. This paper reviews recent advancements in AI applications within cancer research, focusing on early detection through computer-aided diagnosis, personalized treatment strategies, and drug discovery. We survey AI-enhanced diagnostic applications and explore AI techniques such as deep learning, as well as the integration of AI with nanomedicine and immunotherapy for cancer care. Comparative analyses of AI-based models versus traditional diagnostic methods are presented, highlighting AI’s superior potential. Additionally, we discuss the importance of integrating social determinants of health to optimize cancer care. Despite these advancements, challenges such as data quality, algorithmic biases, and clinical validation remain, limiting widespread adoption. The review concludes with a discussion of the future directions of AI in oncology, emphasizing its potential to reshape cancer care by enhancing diagnosis, personalizing treatments and targeted therapies, and ultimately improving patient outcomes.

## 1. Introduction to AI in Cancer Research

Cancer is a major global health challenge, accounting for nearly 10 million deaths in 2022 [1]. An estimated 20 million new cancer cases were reported worldwide, with lung, breast, colorectal, liver, and stomach cancers being the most common. According to the International Agency for Research on Cancer, cancer incidence is projected to rise to 35 million cases by 2050 [1]. It is estimated that 70% of these deaths occur in low-to-middle-income countries, with mortality expected to triple in low-income countries by 2050 compared to high-income countries [1]. Despite significant advancements in medical research, many cancers remain challenging to detect in their early stages, often resulting in delayed diagnoses and poorer outcomes [2,3,4,5]. Enhancing early and accurate detection, combined with personalized treatment approaches, is important for improving survival rates and quality of life for cancer patients worldwide [6].

**Figure 1:**
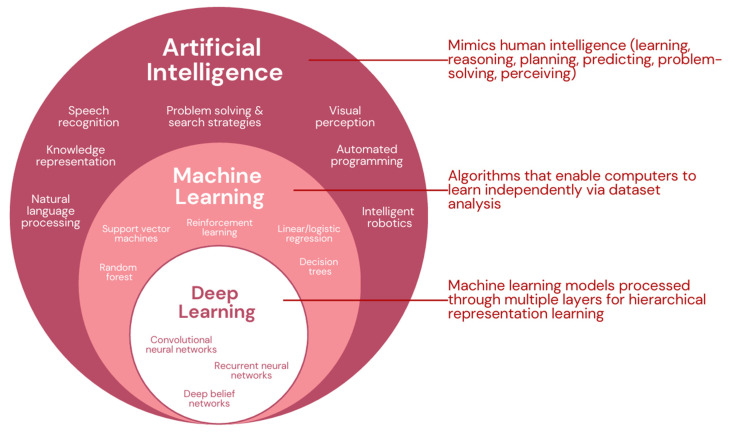
AI classification with examples of subcomponents (adapted from [16,17]).

Artificial intelligence (AI) is defined as the ability of a machine or system to simulate human intelligence, such as learning, reasoning, planning, predicting, problem-solving, and perceiving. Examples of AI subfields include machine learning (ML), deep learning (DL), evolutionary algorithms, and natural language processing (NLP) (Figure 1) [7]. Subfields like ML and DL are particularly influential, with ML enabling autonomous learning from datasets and DL using neural networks to identify patterns through layers of abstraction [7,8,9]. In oncology, supervised ML methods, such as support vector machines and random forest algorithms, have been used for tumor classification and prognosis prediction by analyzing patterns in existing datasets to generate data-driven predictions [10]. Subsets of DL like convolutional neural networks (CNNs) have been employed in radiology studies to investigate their impact on tumor detection in imaging [11]. Recurrent neural networks, DL models that can analyze sequential patterns in data (e.g., speech, text), have been increasingly used in NLP applications, such as extracting clinically relevant information from cancer pathology reports [12]. In recent years, the application of these methods has positioned AI to be a powerful tool for early detection, treatment selection, and personalized patient care [13,14]. In 2020, a study found that the Food and Drug Administration (FDA) is increasingly approving AI medical devices and algorithms, particularly those based on ML, at an increasingly rapid pace [15].

AI-based tools have significantly advanced cancer diagnostics, often matching or surpassing human experts in sensitivity [11,18,19]. One study evaluating AI-assisted cytology found it was 5.8% more sensitive for detection of cervical intraepithelial neoplasia grade 2+ than manual reading, though there was a slight reduction in specificity [18]. CheXNeXt, a CNN designed to detect lung pathologies in chest x-rays, demonstrated 52.3% greater sensitivity in identifying masses and 20.4% greater sensitivity in detecting nodules compared to board-certified radiologists, while maintaining comparable specificity [11]. Incorporating tools like these into clinical practice can streamline diagnostics and expand access to pathologic and radiologic interpretation. This has global significance, as the World Health Organization estimates that 4 billion people lack access to medical imaging interpretation [11]. Moreover, disparities in the cancer burden are projected to continue increasing across countries, age, and sex [20].

The rise of big data has further expanded AI’s impact, paving the way for more accurate predictive and prognostic models that have the potential to significantly enhance clinical decision-making. For instance, The Cancer Genome Atlas, which contains extensive molecular profiles of over 11,000 human tumors from 33 different cancer types, has been leveraged by ML and DL algorithms to generate multimodal (genomics, pathomics, radiomics, etc.) prognostication across a wide range of cancers [21,22]. AI can contribute to personalized medicine by predicting individual responses to chemotherapy, radiation, and surgery, with AI-based approaches already being developed to identify patterns in radiotherapy response using predictive models based on imaging biomarkers [23].

As AI continues to evolve, its application in oncology has the potential to revolutionize cancer care. This review explores the expanding role of AI in oncology through its use in screening, diagnosis, treatment, and personalized medicine, while also addressing its limitations and future directions.

## 2. Applications of Artificial Intelligence in Cancer Diagnosis

Computer-aided diagnosis (CAD) systems have shown significant promise in cancer diagnosis, particularly in the detection, characterization, and monitoring of tumors [24]. CAD systems help physicians interpret medical images by quantitatively analyzing the likelihood of malignancy in suspicious lesions [25,26]. CAD systems can be differentiated into computer-aided detection systems (CADe) and computer-aided diagnosis systems (CADx). CADe systems detect potential abnormalities but do not provide radiological details, leaving interpretation to the radiologist [26]. Conversely, CADx systems serve as a decision aid for radiologists to characterize findings from radiological images identified either by a radiologist or a CADe system and do not yet have a good level of automation [26]. Nevertheless, CAD systems have been shown to improve physician interpretations of images in terms of accuracy in detection and productivity in time spent reading and interpreting images [27,28,29,30,31]. For instance, numerous prospective, multicenter studies have found that real-time use of CADe tools during colonoscopy leads to improved adenoma detection and other related performance metrics [32]. While CAD systems continue to improve, further research is still necessary before CAD systems may function as standalone detection and diagnosis clinical systems [26].

Beyond CAD systems, integrating AI with structured, unstructured, and multimodal fusion data has emerged as a pivotal advancement in cancer diagnostics. Healthcare is inherently multimodal, with structured data including electronic health records (EHRs) and lab results, and unstructured data encompassing clinical notes and imaging reports. In many applications of medicine, the integration, or fusion, of different data sources is necessary for effective clinical decision-making. For oncology, combining modalities like radiology, histology, genomics, and EHRs provides a richer understanding of a patient’s condition [33]. Multimodal fusion data integrates these diverse data modalities, each providing a different perspective on a clinical question, to enhance diagnostic and prognostic accuracy, bringing AI closer to clinical practice. Ultimately, multimodal data fusion aims to extract and combine complementary, contextual information across different modalities for better decision-making [33]. Examples of multimodal fusion data include the integration of different imaging modalities, such as fusion of positron emission tomography (PET) and computer tomography (CT) scans in lung cancer detection, and fusion of magnetic resonance imaging (MRI) and ultrasound images in prostate cancer classification [34,35]. For instance, a supervised CNN has been developed to spatially fuse modality-specific features from PET and CT, achieving superior tumor detection accuracy (99.29%, *p* < 0.05) and a higher Dice score (63.85%) compared to traditional fusion and segmentation methods, illustrating a concrete pathway for AI-enabled PET/CT analysis in clinical workflows [34]. Genomics information has also been used in tandem with histology for improved survival prediction in multiple types of cancer [36].

Multimodal AI models that combine EHR and imaging data generally outperform single modality models in disease diagnosis and prediction [37]. These models offer more robust and accurate diagnostic and prognostic capabilities, aiding in the discovery of novel biomarkers and therapeutic targets [33].

A systematic review of AI-based diagnostic tools that have already obtained official FDA approval found that a vast majority of the approved tools were related to cancer diagnostics, with the largest number of AI devices in breast, lung, prostate, and colorectal cancers, supporting the role of AI in this field [38]. Another systematic review of AI tools for breast cancer detection demonstrated that DL techniques have achieved accuracies exceeding 96%, outperforming conventional ML methods [39]. Currently, lung cancer diagnosis relies mainly on manual pathology analysis, but the low efficiency and subjective nature of manual film reading can lead to misdiagnoses or omissions [40]. Furthermore, current clinical practice of early lung cancer screening using CT scans of the chest is a time-consuming and relatively subjective process that is prone to inter-observer variability [40]. AI-assisted diagnostic systems have already shown significant value for lung cancer diagnosis in terms of improving diagnostic sensitivity of early lung cancer and assisting physicians to screen early lung cancer more effectively and quickly [40]. For example, a meta-analysis of AI algorithms for lung cancer diagnosis has shown a combined sensitivity and specificity of 87%, significantly reducing misdiagnosis rates compared to manual pathology section analysis [40]. However, one limitation of AI-assisted diagnosis involves a high level of heterogeneity among studies, as different algorithms have different diagnostic outcomes [40]. An international study demonstrated that a validated AI system had a superior AUC (0.91) compared to radiologists (0.86) and detected more cases of Gleason grade group 2 or greater cancers at the same specificity [41]. Urban et al. reported that their AI-based CADe system improved the detection of colorectal polyps, with sensitivity and specificity rates of 97% and 95%, respectively, outperforming human endoscopists [42]. It is evident that AI-based diagnostic tools have been continuously refined over the past decade, and their diagnostic performance has been demonstrated to match or even surpass that of human experts in multiple different cancer types [19,43,44].

AI-based diagnostic tools enhance accuracy, efficiency, and early cancer detection, improving patient outcomes. Although most studies evaluating AI applications in oncology have not been vigorously validated for reproducibility and generalizability, AI offers an objective way to incorporate complementary information and clinical context from diverse data for improved predictions (Table 1) [33].

## 3. Deep Learning and Artificial Intelligence in Oncology

As a subset of ML, DL employs neural networks to analyze and interpret large datasets. Information is transmitted sequentially between network layers as weighted summations [45]. A simple multi-layer perceptron network with input, hidden (not directly visible from the input or output—only the network itself processes it), and output layers can scale to deeper architectures with multiple hidden layers [46]. DL models can be tailored to specific applications and data types.

DL models, such as convolutional neural networks (CNNs), outperform traditional diagnostics with high accuracy in medical image analysis. These models analyze raw data through convolutional and pooling operations, segment whole slide images, and normalize staining as part of preprocessing. AI-based systems train and validate models, optimizing performance across datasets [47]. Software based on such models has achieved high precision in differentiating between benign and malignant lung cancer tissue, increasing early cancer detection accuracy to 96.07% [48]. Training these models on large, well-annotated datasets enables more precise classification of images based on various pathologies [49]. A CNN-based model, DenseNet121, demonstrated 99.4% accuracy in classifying seven cancers, including breast, colon, and lung cancer [50]. Beyond CNNs for automated abnormality detection in medical imaging, recurrent neural networks (RNNs) are now used for time-series data analysis in oncology, potentially enabling longitudinal tracking of treatment responses [51]. Custom neural networks, trained on relevant datasets, use attention mechanisms to highlight key medical features.

Beyond image analysis, AI can also process key oncological markers, including the tumor’s molecular profile and its interactions with the surrounding environment. The tumor microenvironment (TME) is a complex ecosystem consisting of a plethora of reactive species and organelles, as well as both immune and non-immune cells [52]. New AI techniques analyze tumor interactions at the cellular level, providing spatial and quantitative data using metabolomics [53]. TME models help decode patient-specific variations, enabling personalized treatment and more accurate therapy response analysis [47]. In a recent study, a DL model using Mask R-CNN successfully segmented and classified macrophage nuclei from HE-stained lung adenocarcinoma images, while a complementary ML approach based on cell morphology achieved 90% accuracy in distinguishing M1 and M2 macrophage phenotypes from naïve macrophages and monocytes [54,55]. CNN-based models have also demonstrated strong potential in cellular analysis, achieving over 95% accuracy in cancer cell capture within milliseconds using flow cytometry data, and reaching up to 90% accuracy in classifying benign and malignant urothelial cells using EfficientNet B6 and ArcFace [56,57]. While models like the one developed by Chawan et al. can distinguish cholangiocarcinoma cells based on morphology, reliance on shape alone may lead to misclassification of damaged or irregular cells, highlighting the need for more integrated feature approaches [47,58]. Additionally, the combination of MRI data with genetic profiles through multimodal analysis has already proven effective in grading gliomas and identifying drug resistance mechanisms [59].

Molecular profiles and TMEs provide critical insights into tumor behavior and progression [60]. AI-based models, which can integrate data from metabolomics, genomic sequencing, and imaging, now replace traditional gene sequencing labs by efficiently characterizing molecular profiles [61]. AI models can enhance predictive accuracy for post-trauma complications by processing large, annotated datasets—such as biomarker profiles from trauma patients—revealing early immune response patterns linked to nosocomial infections and prolonged critical illness [62]. Advanced DL techniques, like graph neural networks, use annotated graphs to represent molecular data. When combined with physics-informed ML, these networks improve drug discovery accuracy [63]. A recent AI model, named The Clinical Histopathology Imaging Evaluation Foundation (CHIEF), identifies key genetic mutations and predicts tumor responses to targeted therapies. It also generates heatmaps of tumor–microenvironment interactions for pathologist analysis [64]. The accuracy of this tool underscores the growing, self-propagating predictive power of AI in cancer research and treatment.

## 4. AI in Nanomedicine and Nano-Oncology: Enhancing Cancer Treatment and Drug Delivery

AI has advanced progress in nanomedicine and nano-oncology by driving innovation in drug delivery, diagnostics, and personalized treatment. ML models in particular are able to optimize the use of nanocarriers, refine therapeutic options, and improve real-time patient monitoring. Integrating AI with nanotechnology addresses challenges in drug formulation, delivery kinetics, and biomarker tracking, improving treatment efficacy while minimizing adverse effects.

### 4.1. AI-Driven Optimization of Nanomedicine in Drug Delivery Systems (DDSs)

AI plays a key role in the rational design and high-throughput development of nanomaterial-based drug delivery systems (DDSs), expediting the production of nanoformulated drugs with predefined functionalities [65]. ML models can analyze expansive datasets to predict drug delivery kinetics, optimize nanocarrier properties, and enhance the stability and scalability of nanomedicines [66,67], which reduces systemic toxicity and may ultimately improve patient outcomes. AI overcomes longstanding challenges in nanomedicine design by analyzing complex experimental data. High-throughput experimentation, data science techniques, and automation are now essential for developing DDSs with tailored functionalities [65]. This integration is referred to as “The Fourth Paradigm of Scientific Research”, where data-driven methodologies enable more effective and scalable drug formulations. 

Additionally, AI can assist in the characterization of nanoparticles by using high-throughput transmission electron microscopy (TEM) analysis [68]. AI-driven image processing techniques, using genetic algorithms, allow for the precise classification of nanoparticle morphology to ensure quality control in nanomedicine production. This approach allows researchers to analyze over 150,000 nanoparticles with an accuracy of 99.75%, greatly optimizing the design of DDSs. In the context of personalized nanomedicine, AI has been used to improve nanocarrier-based DDSs for prostate cancer therapy [69]. Promising advancements in prostate cancer diagnostics are demonstrated by the FDA-approved Paige Prostate model, which uses multiple instance learning trained on 12,132 whole slide images and achieved an AUC of 0.99 on trial data and 0.93 on an external validation set of approximately 12,000 slides [69,70]. Its implementation increased pathologists’ diagnostic sensitivity from 74 % to 90 %, with further validation on 600 patient samples and 1876 prostate core biopsy whole slide images. In parallel, recent studies have advanced AI-driven nanocarrier systems: one study reported a 40 % improvement in PSMA-targeted delivery in mice, while another achieved a 30 % reduction in off-target toxicity using DL predictions validated in 15 prostate cancer patients [71,72]. AI further enhances nanocarrier design by predicting drug delivery kinetics and enhancing ligand-targeting mechanisms, which are essential for overcoming tumor heterogeneity and drug resistance. 

### 4.2. AI-Powered Sensors for Cancer Diagnosis and Monitoring

AI-powered nanosensors improve cancer diagnostics by offering high-precision data acquisition and analysis in real-time. These sensors can integrate AI-driven algorithms to process multi-dimensional data, enhance early detection, track tumor progression, and assess treatment response with greater accuracy than traditional methods [73,74]. AI also significantly improves optical imaging techniques, such as photoacoustic imaging, optical coherence tomography, and fluorescence imaging, strengthening the ability to visualize TMEs [74]. In more complex diagnostic challenges, such as cancers of unknown primary origin—which account for 1–2% of cases and are associated with a poor median overall survival of 2.7–16 months—DL tools like Tumor Origin Assessment via Deep Learning (TOAD) have shown clinical utility. Trained on over 22,000 whole-slide images, accurately identifying the tumor origin in 83% of known cases and included the correct diagnosis among its top three predictions 96% of the time; in 317 cases of unknown primary origin, it matched the pathologist’s report in 61% and the top three in 82% [75]. Additionally, in gastric cancer, AI-aided endoscopy demonstrated a 100% detection rate, outperforming expert endoscopists who achieved 94.12% accuracy [76,77]. In a study by Yamada et al., an AI model trained on colon capsule endoscopy images achieved an AUC of 0.902, with 79.0% sensitivity and 87.0% specificity for detecting colorectal neoplasias; however, its performance was limited by factors such as poor image quality, orientation issues, and lesion variability, underscoring the current constraints of AI in endoscopic diagnostics [74,78].

Additionally, AI-powered sensors can integrate multi-omics profiling to combine genomic, epigenomic, transcriptomic, and proteomic data and identify disease-specific biomarkers [73], aiding in tumor behavior prediction and personalized treatment planning. This allows for a deeper understanding of the molecular changes associated with cancer progression and treatment response. Beyond imaging and biomarker analysis, AI can also facilitate predictive analytics in cancer management. AI models may forecast treatment responses and disease progression by analyzing historical and real-time data [79,80]. For instance, Duanmu et al. developed a DL model incorporating spatial attention and immunohistochemical biomarkers Ki67 and PHH3 to predict pathological complete response to neoadjuvant chemotherapy in triple-negative breast cancer, achieving 93% accuracy in a cohort of 73 patients [80]. Through use of AI’s predictive capability, we can adapt our cancer therapies to ensure that treatments evolve in response to changes in tumor dynamics.

### 4.3. AI in Personalized Nanomedicine and Therapeutic Synergism

Algorithms such as deep reinforcement learning and generative adversarial networks can enhance the design of nanomedicine, drug targeting, and dosing strategies [81]. They are able to predict drug interactions, optimize multi-drug regimens, and refine therapeutic synergy in cancer treatment. Deep neural networks and CNNs also contribute to drug discovery by identifying optimal nanocarrier compositions and delivery mechanisms. Models like CURATE.AI enable real-time dosing adjustments and toxicological risk predictions, ensuring that each patient receives a highly individualized treatment plan with maximum therapeutic efficacy and minimal side effects [82].

Nanomedicines are playing an increasingly important role in medical care, with over 80 FDA-approved nanomedicines reaching the market since 1989 [83,84]. Many nanomedicines are in different stages of clinical development, underscoring their growing role in healthcare [85,86]. An early example is Ontak, a targeted protein-based nanoparticle that achieved a 63.3% overall survival rate when combined with CHOP chemotherapy in peripheral T-cell lymphoma, compared to 32–35% with CHOP alone, without notable myelosuppression or organ toxicity [86]. AI has further accelerated advancements in single-molecule real-time sequencing and nanopore sequencing, significantly improving DNA analysis accuracy for cancer diagnostics. AI-driven nanosensors, derived from nanomedicines, may offer superior sensitivity and lower detection limits than traditional biosensors, making them a promising tool for early disease detection and personalized treatment planning [87,88]. AI also demonstrates potential in nanotheranostics, where nanotechnology-based imaging and therapy are combined into a single platform [69]. By integrating AI with nanotechnology, explainable AI models and multi-modal data fusion can further enhance precision oncology. Furthermore, AI automates the analysis of clinical trial data, ensuring that potential safety risks are identified early. AI frameworks like DeepDR and SNF-CVAE enhance drug repurposing by uncovering new therapeutic applications for existing nanomedicines, reducing development costs and expediting clinical translation [81]. As with any application of AI, regulatory and data accessibility challenges exist. However, AI-driven innovations in design and immune system interactions continue to revolutionize the field of nanomedicine [69].

## 5. Artificial Intelligence in Immunotherapy

AI has also been studied as an emerging support modality for immunotherapeutic cancer interventions. Immunotherapeutic therapies include monoclonal antibodies, adoptive cell transfer, vaccinations, and oncolytic viruses [89,90,91], all with the purpose of stimulating or modifying the host immune system to seek and destroy malignant cells. AI can be leveraged to support several of these already existing interventions. For example, neural networks have been developed to predict peptide binding affinity of major histocompatibility complex (MHC) molecules for neoantigen recognition and more personalized antibody design [92]. AI has also been utilized for RNA sequencing to describe the TME at a polypeptide level, which therefore lends itself to a more personalized understanding of therapeutic interventions [93]. Likewise, computing models are able to incorporate radiology, pathology, genomics, and clinical data to not only optimize but also predict patient clinical outcomes [94].

### 5.1. Checkpoint Inhibition

These tools generally aim to predict the efficacy of biomolecular blockade mechanisms. For example, PD1/PD-L1 inhibitors suppress the immunomodulatory interaction between PD-L1, often expressed on the surface of tumor cells, with PD1 on T cells [95,96]. The ELISE model, developed in 2022, integrates neural networks and patient data to predict PD-1/PD-L1 inhibitor efficacy, achieving an AUC of 88.86% in metastatic urothelial cancer [97]. This therapy is a form of immune checkpoint blockade, using molecular decoys to inhibit tumor–immune cell interactions [98]. Models such as ELISE utilize genomic, molecular, demographic, and clinical information to better predict immune checkpoint blockade effectiveness [99].

### 5.2. AI-Driven Models

AI tools improve immunotherapy via the identification of predictive biomarkers for immune checkpoint inhibitors (ICIs), thus enabling more personalized therapeutic approaches. ML algorithms can analyze gene expression and immune microenvironment biomarkers. Identification of oncogenes, tumor suppressor genes, and immune markers such as MYC, BCL2, TP53, PD-L1, PD-1, CD68, and CD163 guides understanding of interactions between tumor cells and immune cells [100]. Spatial analysis of tumor-infiltrating lymphocytes enables classification of immune phenotypes as either inflamed, immune excluded, or immune desert, which have been found to each separately correlate with ICI response in patients with non-small cell lung cancer [101]. Inflamed tumors have demonstrated better response rates to ICI treatment relative to non-inflamed tumors. For example, in biliary tract cancers, anti-PD-1 therapy led to a 27.5% objective response rate in inflamed tumors compared to 7.7% in non-inflamed tumors (*p* < 0.001) [102]. AI also aids in peripheral immune cell profiling, identifying predictive biomarkers such as PD-L1 expression on monocytes and CD8 T cells, which impact treatment decisions [103]. Furthermore, AI networks develop genomic mutation signatures to predict prognosis and response to ICIs in gastrointestinal cancers, demonstrating AUC values from 0.8417 to 0.875 [103]. By utilizing multi-omics data, spatial immune profiling, and advanced predictive models, AI tools will likely lead to better outcomes and fewer unnecessary treatments.

### 5.3. Radiographic and Non-Molecular Mechanisms

There is use for AI in the realm of cancer immunotherapy at the anatomical level as well. One 2019 study utilized an AI program to analyze contrast-enhanced CT imaging to develop an ML biomarker for non-small-cell lung cancer [104]. This algorithm took lesion texture, shape, intensity, and spatial heterogeneity into account and used these to generate a prediction of whether or not the tumor would respond to anti-PD1 immunotherapy. There is vast potential for radiographic imaging to be leveraged in predicting immunotherapeutic response, which can help prevent patients from undergoing treatments that are not likely to yield positive results [105]. AI can also help researchers and clinicians better understand the tumor microenvironment and therefore make educated predictions regarding patient outcomes. ML models can also process PET scans, which highlight metabolically active tissues, to predict patient prognosis and characterize tumor phenotypes based on biochemical characteristics [106]. AI tools can integrate multiple modalities, including radiology, pathology, and genomics, to help support more personalized treatment for patients. Recent academic endeavors have combined radiomics, pathomics, and genomic data to predict PD-L1 expression, tumor mutation burden, and tumor microenvironment in lung cancer patients. Better understanding of these biomarkers is crucial for assessing and predicting immunotherapy responses [107]. Table 2 summarizes recent AI advancements in deep learning, nanomedicine, and immunotherapy. 

## 6. Social Determinants of Health and AI in Cancer Care

Cancer affects all backgrounds, and AI-driven healthcare must account for social determinants of health (SDOH). The World Health Organization defines SDOH as “non-medical factors that influence health outcomes”, such as “the conditions in which people are born, grow, work, live, and age”, as well as broader social and economic policies [115]. A scoping review identified three main themes regarding AI’s impact on health equity in oncology: the potential for AI to reduce healthcare disparities, concerns about bias in AI technologies, and the role of AI in examining both biological and social determinants of health [116], which this segment will explore.

### 6.1. Financial Toxicity

In the United States, cancer is the second leading cause of death, with up to 75% of cases linked to SDOH; poverty is among the strongest predictors of mortality [117]. Financial toxicity is an especially significant burden. A study of 9.5 million cancer cases found that 42.4% of patients depleted their life savings within two years, and 38.5% remained insolvent after four years [118]. Those at the highest risk included women, Medicaid recipients, uninsured, retirees, older adults, and individuals with lower incomes [116]. The economic impact of health disparities in the United States is devastating, contributing to USD 93 billion in excess medical care costs and USD 42 billion in productivity losses from related premature deaths per year [119]. By 2030, national cancer-attributable medical costs of care are projected to reach USD 246 billion [120]. Beyond financial hardship, the stress of mounting medical expenses can exacerbate emotional distress and health outcomes [121,122]. Addressing SDOH in cancer care is not only beneficial but cost-effective. A systematic review of interventions targeting SDOH to improve breast, cervical, and colorectal cancer screening found that such programs effectively serve vulnerable populations. With a median intervention cost of USD 3120 per quality-adjusted life year (QALY)—well below the conservative USD 50,000/QALY threshold—these initiatives present a compelling case for investment [117]. Several studies have shown that financial toxicity in cancer patients can be predicted prior to treatment using algorithms. For instance, one study involving breast cancer patients used patient-reported data and clinical outcomes to predict financial toxicity [122]. Another study with female surgery patients identified key factors such as neoadjuvant therapy and low credit scores as contributors to financial toxicity, while a third study with lung cancer patients stratified moderate versus severe financial toxicity after surgery [123,124]. Given the staggering financial costs associated with cancer care mentioned previously, a predictor like this could help support patients in advance by enabling healthcare providers to offer timely interventions, such as financial counseling, assistance programs, or adjustments to treatment plans that prioritize cost-effective options. Early identification of financial strain could also facilitate improved access to resources and better coordination of care, ultimately reducing the risk of delayed treatments or abandonment of care due to financial hardship.

### 6.2. Harvesting Unstructured Data Potential

A significant portion of healthcare documentation exists as free text, whether in patient charts or communications. This vast pool of unstructured data holds potential for predicting clinical outcomes and improving cancer care. AI-driven models have demonstrated an 80% accuracy rate in predicting cancer patients’ disease trajectories by analyzing free text in EHRs [125]. Extending this approach to SDOH could enhance predictive analytics, yet research on NLP-based SDOH extraction remains limited. Studies show that key determinants, like smoking, education, and employment status, are more often documented in clinical narratives than in structured data, exposing gaps in EHRs [117,126]. In a similar study, ten SDOH categories were documented in more than 70% of cancer patients, whereas nine other categories—such as financial constraints, living conditions, physical activity, and transportation—had a lower extraction ratio (<70%), suggesting gaps in how these factors are recorded in EHRs [126]. Black patients had over 10% higher documentation rates than other groups for certain SDOH categories, including abuse, financial constraints, and living conditions [124]. Another study explored the potential of large language models to extract SDOH information from narrative text in EHRs to improve clinical care and research. Text-extracted data identified 91.8% more patients with adverse SDOH than structured diagnostic codes [127]. These studies highlight the wealth of information captured in free-text clinical notes that may not be immediately salient but hold significant clinical value. For example, if a provider documents that a patient misses follow-up appointments due to lack of transportation or childcare, NLP could identify and flag this as a health risk factor. Moreover, this research underscores potential biases, such as the under documentation of certain SDOH or the reinforcement of broader social disparities affecting vulnerable populations.

### 6.3. Key SDOH Considerations and Areas for Further Research

ML has also been shown to enhance patient navigation, improving health outcomes in marginalized populations [128]. Additionally, a study using ML models to predict cancer risk identified age, race, sex, and housing status as key factors, while another large-scale study found that SDOH such as neighborhood crime index, home values, annual income, and wealth index were significant predictors of unplanned 30-day hospital readmissions [129,130]. Another review focusing on US-based cancer screenings (breast, cervical, colorectal, and lung) found that SDOH interventions related to healthcare access and quality were most common. However, other SDOH, such as educational, social/community, environmental, and economic factors, were less frequently addressed [131]. This review also emphasized the need to expand research beyond individual-level SDOH to include structural, community, and healthcare system levels [125]. While these broader factors are crucial, it is equally important to continue prioritizing individual-level SDOH, as existing AI models and research methodologies often fail to accurately capture personal and demographic nuances—particularly for marginalized populations [132,133]. For example, resource limitations lead to gaps in documenting patients’ biological, social, and health behaviors, potentially leading to confounding issues in statistical analyses or misclassification errors in ML [117]. Furthermore, AI technologies have demonstrated bias, as illustrated by IBM’s facial recognition systems being 11% to 19% less accurate for recognizing black men and 34% less accurate for black women [134]. Another study found racial disparities in healthcare risk prediction algorithms, where black patients were often sicker than white patients at the same risk score, leading to unequal healthcare allocation [131].

While the integration of AI in healthcare offers immense promise, the ethical and efficient use of AI remains an ongoing discussion. A recent scoping review identified significant gaps, particularly regarding the ethical challenges of AI technologies in low- and middle-income countries [116]. While AI bias is a well-known issue, actionable solutions are less frequently discussed. To address this, we advocate for rigorous audits of training data, the development of bias-correction algorithms, and the implementation of transparent decision-making frameworks. Effective research on SDOH requires extensive sensitive health data. To minimize bias and ensure fair outcomes, AI research must prioritize diverse, representative datasets, particularly those encompassing minorities and “data-impoverished” groups [116]. Moreover, achieving AI’s full impact in healthcare will necessitate a collaborative effort from clinicians, AI researchers, patient advocacy groups, health equity scholars, government agencies, and industry stakeholders [135]. As AI continues to shape the future of medicine, a commitment to inclusivity, transparency, and ethical responsibility will be essential in driving meaningful and lasting improvements in health equity.

## 7. Conclusions, Challenges, and Future Directions

Overall, we are optimistic about integrating AI with CAD systems, personalized treatment strategies, and drug discovery. However, challenges such as data standardization, model interpretability, and clinical integration must be addressed [136]. A key concern is that DL models often function as a “black box”, making AI predictions difficult to interpret. Enhancing AI explainability is crucial for building trust and ensuring clinical adoption. While AI improves diagnostic accuracy and detects errors overlooked by humans, we advocate for human-in-the-loop models to ensure that AI remains a collaborative tool rather than an autonomous decision-maker. This is especially important in addressing disparities linked to SDOH, where gaps in healthcare access and documentation can impact diagnostic accuracy and treatment outcomes.

Beyond diagnostics, AI is transforming cancer treatment by refining drug discovery, optimizing nanocarrier design, and improving predictive analytics for personalized medicine [81]. AI-powered CAD systems and deep learning models are enhancing early cancer detection, surpassing traditional imaging in sensitivity and specificity [38,39,42]. AI-driven multimodal analysis is improving risk stratification and treatment selection, leading to more precise and individualized therapies [21,22,23]. In nano-oncology, AI is advancing drug delivery systems to maximize efficacy while minimizing toxicity [65,69], while AI-powered immunotherapy applications are improving biomarker identification and predicting patient responses to immune checkpoint inhibitors [97,103].

Ultimately, AI’s ability to process vast and complex datasets is revolutionizing oncology by enabling earlier detection, more targeted treatments, and improved patient outcomes. As AI research advances, these technologies will not only support clinicians in decision-making but also enhance survival rates and expand access to high-quality cancer care. AI has the potential to significantly impact the future of oncology, provided its advancements are supported by robust clinical validation and real-world application.

## Figures and Tables

**Table 1:** Applications of AI in cancer diagnosis.

Application	Description	Key Findings	References
CAD systems	AI-assisted detection (CADe) and diagnosis (CADx) of tumors via medical imaging.	Improves accuracy and efficiency; CADe enhances adenoma detection in colonoscopy.	[24,25,26,27,28,29,30,31,32]
Multimodal AI Models	Integrates imaging, histology, genomics, and EHRs for enhanced diagnostics.	PET-CT aids lung cancer detection; MRI-ultrasound improves prostate cancer classification.	[33,34,35,36]
AI for Cancer Imaging	AI-based tools enhance accuracy in detecting various cancers.	Breast: >96% accuracy; Lung: 87% sensitivity/specificity; Prostate: AI AUC 0.91 vs. radiologists 0.86; Colorectal: 97% sensitivity, 95% specificity.	[38,39,40,41,42]
AI in Early Detection and Prognosis	AI improves screening, early diagnosis, and predictive modeling.	Matches or surpasses human experts but needs better validation for generalizability.	[33,43,44]

**Table 2:** AI advancements in deep learning, nanomedicine, and immunotherapy.

Application	Description	Models Utilized	Key Findings	References
Deep Learning in Oncology	CNNs and RNNs improve medical imaging, tumor classification, and time-series data analysis.	DenseNet121, GoogLeNet, AlexNet (pre-trained on ImageNet)	DenseNet121 CNN achieved 99.4% accuracy in classifying seven cancers.	[45,46,47,48,49,50,51,108]
TME Analysis	Analyzes tumor interactions at the cellular level for personalized therapy.	AI-enhanced MRI, genomics, SiQ-3D (Single-cell image quantifier for 3D)	AI-enhanced MRI and genomics improve glioma grading and identify drug resistance.	[52,53,59,109]
Molecular Oncology	Integrates genomics, transcriptomics, and imaging to predict tumor progression and therapy response.	CHIEF AI model trained on 15 million unlabeled images	CHIEF AI model predicts tumor mutations and therapy responses.	[60,61,62,63,64]
Nanomedicine and Drug Delivery	Optimizes nanoparticle design, drug delivery systems (DDSs), and treatment efficacy.	AI-based TEM analysis, FakET (trained on synthetic datasets)	AI-based TEM analysis achieved 99.75% accuracy in nanoparticle classification.	[65,66,67,68,69,110]
AI-Powered Sensors	Enhance real-time biomarker detection and tumor tracking.	TriTom (integrates photoacoustic and fluorescence imaging)	Improves photoacoustic and fluorescence imaging for TME visualization.	[73,74,79,80,111]
Personalized Nanomedicine	Refines nanocarrier targeting, drug interactions, and dosing.	CURATE.AI (uses minimal input-output data pairs)	CURATE.AI optimizes therapy for individualized dosing.	[81,82]
Immunotherapy	Predicts immune responses and enhances checkpoint inhibitor therapy.	SCORPIO (trained on clinical data from 1628 patients)	ELISE model achieved 88.86% AUC in predicting PD-1/PD-L1 inhibitor efficacy.	[89,90,91,92,93,94,95,96,97,98,99,112],
Imaging-Based Immunotherapy Response	Analyzes CT/PET scans to predict treatment response.	TME-radiomic models, DCE-MRI, Synthetic Methionine PET	Predicts anti-PD1 therapy response using contrast-enhanced CT.	[100,101,102,103,104,105,106,107,113,114]

## Abbreviations

**Abbreviation**	**Full Term**
AI	Artificial Intelligence
BCL2	B-cell Leukemia/Lymphoma 2 Protein
CAD	Computer-Aided Diagnosis
CADe	Computer-Aided Detection
CADx	Computer-Aided Diagnosis System
CD	Cluster of Differentiation
CNN	Convolutional Neural Network
CT	Computed Tomography
DDS	Drug Delivery System
DL	Deep Learning
EHR	Electronic Health Record
FDA	Food and Drug Administration
ICI	Immune Checkpoint Inhibitor
ML	Machine Learning
MRI	Magnetic Resonance Imaging
MYC	Avian Myelocytomatosis Viral Oncogene Homolog
NLP	Natural Language Processing
PD-1	Programmed Cell Death Protein 1
PD-L1	Programmed Death Ligand 1
PET	Positron Emission Tomography
RNN	Recurrent Neural Network
SDOH	Social Determinants of Health
TEM	Transmission Electron Microscopy
TME	Tumor Microenvironment
TP53	Tumor Protein 53
